# The Values and Perspectives of Organoids in the Field of Metabolic Syndrome

**DOI:** 10.3390/ijms24098125

**Published:** 2023-05-01

**Authors:** Chen Tan, Min Ding, Yun-Wen Zheng

**Affiliations:** 1 Institute of Regenerative Medicine, Department of Dermatology, Affiliated Hospital of Jiangsu University, Jiangsu University, Zhenjiang 212001, China; tanchen@stmail.ujs.edu.cn (C.T.); dingmin@stmail.ujs.edu.cn (M.D.); 2 Department of Medicinal and Life Sciences, Faculty of Pharmaceutical Sciences, Tokyo University of Science, Noda 278-8510, Japan; 3 School of Medicine, Yokohama City University, Yokohama 234-0006, Japan; 4 Center for Stem Cell Biology and Regenerative Medicine, Institute of Medical Science, The University of Tokyo, Tokyo 108-8639, Japan

**Keywords:** metabolic syndrome, pancreatic organoids, β-cells, diabetes, islet regeneration, hepatobiliary organoids, liver fibrosis, liver regeneration, hepatic macrophages

## Abstract

Metabolic syndrome (MetS) has become a global health problem, and the prevalence of obesity at all stages of life makes MetS research increasingly important and urgent. However, as a comprehensive and complex disease, MetS has lacked more appropriate research models. The advent of organoids provides an opportunity to address this issue. However, it should be noted that organoids are still in their infancy. The main drawbacks are a lack of maturity, complexity, and the inability to standardize large-scale production. Could organoids therefore be a better choice for studying MetS than other models? How can these limitations be overcome? Here, we summarize the available data to present current progress on pancreatic and hepatobiliary organoids and to answer these open questions. Organoids are of human origin and contain a variety of human cell types necessary to mimic the disease characteristics of MetS in their development. Taken together with the discovery of hepatobiliary progenitors in situ, the dedifferentiation of beta cells in diabetes, and studies on hepatic macrophages, we suggest that promoting endogenous regeneration has the potential to prevent the development of end-stage liver and pancreatic lesions caused by MetS and outline the direction of future research in this field.

## 1. Introduction

With the progress of modernization and the improvement of living standards, more and more people are suffering from metabolic syndrome. The main characteristic components of metabolic syndrome (MetS) include high blood pressure, increased triglycerides, hyperglycemia, poor HDL cholesterol, and obesity (especially central obesity). Insulin resistance and hyperinsulinemia brought on by obesity, particularly central obesity, define its pathophysiology [1]. Its pathology is characterized by insulin resistance and hyperinsulinemia caused by obesity, especially central obesity [2]. The development of MetS has led to the emergence of many diseases, such as diabetes, cardiovascular disease, fatty liver, and certain cancers [3,4], which are becoming increasingly dangerous global health issues. Many contributing factors and mechanisms have been proposed, including insulin resistance, adipose tissue dysfunction, chronic inflammation, oxidative stress, circadian rhythm disorders, effects of gut microflora, genetic factors, and maternal influences [5]. These mechanisms are redundant and complex, and there are still no effective intervention strategies for the prevention and treatment of diabetes and cardiovascular disease caused by MetS.

MetS is a comprehensive and holistic disease that affects multiple systems in the human body due to its complex causes and effects. Previous studies on metabolic syndrome have mostly used animal and in vitro 2D models, which have their limitations [6,7]. Due to its single monolayer cell plane, 2D cell culture is distant from the actual human body in terms of physiology, biochemistry, and biomechanics, especially in the study of comprehensive diseases such as metabolic syndrome, and is unable to elaborate well on the disease mechanism of the interactions between cells. Cell lines usually do not respond well to glucose stimulation and cannot mimic the interaction between β-cells and other types of cell interactions, which are crucial in islet function and diabetes pathogenesis. In contrast to humans, animal models differ substantially in metabolic pathways, immune systems, and other microenvironments [8]. In many cases, drug candidates developed from mouse trials either had no effect on obesity in humans or, when they did, resulted in significant adverse side effects that were only seen in humans. A particularly notorious example is the appetite-suppressant drug fenfluramine. After successful trials in mice, fenfluramine was approved in the U.S. in 1973, however, in 1997, one-third of patients taking the drug were diagnosed with pulmonary hypertension and heart valve disease [9,10].

In terms of metabolic functionality, gene and protein expression, and microstructure, 3D organoids are more similar to primary organs than previous research methods. This makes organoids a more effective research tool for advancing our understanding of MetS. There are three defining characteristics of organoids: (1) multiple cell types that contain the organ; (2) structural features that compose the organ; and (3) functions that the organ performs [11]. Thus, organoids mimic cellular interactions more comprehensively than 2D-cultured cellular models due to the organoids’ characteristic of multiple cell types. In addition, compared to organ models taken directly from the human body, organoids are more readily available and can be personalized; in contrast to animal models, organoids can originate directly from humans, so there is no need to extrapolate findings from model animals to humans [11,12].

However, organoid technology is still in its infancy and has a long way to go before it can be utilized effectively in disease modeling and regenerative medicine [13]. With the combination of organoid and CRISPR-based gene editing technology, 3D scaffolding and 3D bioprinting technology, a microfluidic device system, organoid chip technology [14], and further refinement of in vitro organoid research, there is hope to create an in vitro research system that gradually approximates the real environment in the human body, providing more accurate and convenient technical support for various diseases, not only MetS.

In this review, we will discuss the significance and potential applications of pancreatic and hepatobiliary organoids in the field of MetS based on their current research status. So far, pancreatic and hepatobiliary organoids have more mature culture protocols and are closely related to MetS.

There has been much evidence that exercise and dietary strategies, to some extent, can reverse insulin resistance, blood pressure, and lipid levels in metabolic syndrome [15,16,17]. Furthermore, in 2021, an observational study also noted that in patients with non-alcoholic steatohepatitis and obesity, bariatric surgery was associated with a significantly lower risk of serious adverse liver outcomes and major adverse cardiovascular events compared to nonsurgical treatment [18]. Therefore, this review also addresses the following question: Based on the research and application of hepatobiliary and pancreatic organoids, is the search for effective external interventions to promote endogenous regeneration a viable strategy for preventing the progression of hepatobiliary and pancreatic MetS to end-stage?

## 2. Pancreatic Organoids

The metabolic syndromes and pancreatic functions have an interdependent and interactive relationship. On the one hand, free fatty acids (FFA) in MetS stimulate body cells as a result of increased production of highly active reactive nitrogen clusters (RNS) and reactive oxygen species (ROS), which in turn initiate oxidative stress mechanisms. These reactive molecules can directly oxidize and damage lipids, proteins, and DNA, as well as act as mobile molecular signals, activating a variety of stress-sensitive intracellular signaling processes that are closely linked to impaired β-cell function [19,20]. On the other hand, the MetS-induced damage to β-cells decreases the function of insulin secretion, which is responsible for glycogen synthesis, protein synthesis, promoting GLUT4 translocation and adipogenesis, as well as hepatic gluconeogenesis and inhibiting apoptosis. Consequently, the decrease in insulin secretion exacerbates the metabolic disorder and promotes the progression of MetS [21].

Multiple cells have been shown to contribute to the development and insulin-secreting function of β-cells, including α-cells, δ-cells, and polypeptide (PP) cells, different subtypes of β-cells [22], and the ecological microenvironment in which they are located. Therefore, a single study of human planar cells cannot generalize the effect of MetS on pancreatic insulin secretion. On the other hand, in human, the stochastic behavior of insulin-releasing β-cells may reflect their unique cytoarchitectonic alignment along the laminar epithelial sheet. Rodents are not an ideal model for studying human pancreatic β-cell dysfunction due to differences in cellular arrangement morphology and the ratio of individual cells within human islets [8,23,24,25].

In addition to β-cells, the endocrine pancreas has α-cells, which produce glucagon; δ-cells, which produce somatostatin; and PP cells, which produce the pancreatic polypeptide. These hormones regulate blood glucose levels in precise balance with insulin and promote energy metabolism [6,26,27]. Therefore, the study of MetS also requires that the impact of other endocrine cells in pancreatic islets on human metabolism be considered, and the generation of homogenous, intact, scaled-up islets in vitro may facilitate the study of MetS. Stem cell-derived organoid technology offers the possibility of achieving this purpose.

### 2.1. The Secretion of Insulin Requires the Cooperation of Different Cells

Blood glucose homeostasis is an important component of metabolic homeostasis, and it is known that insulin is the most important hormone regulating blood glucose in the body [21]. Pancreatic β-cells are the only insulin-producing cells in humans and almost all other vertebrates.

However, multiple types of β-cells are involved in insulin secretion, and many studies have shown that β-cells in pancreatic islets are heterogeneous [28]. Dorrell’s group classified human β-cells into four subpopulations since human β-cells can synthesize different levels of the cell surface proteins ST8SIA1 and CD9. Importantly, the relative abundance of the different subtypes was dependent on metabolic status, with different frequencies observed in T2D. Hence, the diabetic state may lead to a redistribution of β-cell subtypes and even alter the subtype pattern, contributing to the pathogenesis of T2D [29].

Several studies have shown that β-cell heterogeneity has a significant impact on glucose-responsive insulin secretion (GSIS). For example, enteroendocrine L-cells and K-cells release glucagon-like peptide-1 (GLP-1) and glucose-dependent insulinophilic polypeptide (GIP), which enhance GSIS to counteract postprandial hyperglycemic spikes. Studies have shown that GLP-1 recruits a highly coordinated heterogeneous subnetwork of β-cells to enhance GSIS, which is the target of the lipotoxic attack to reduce insulin secretion. Donor body mass index (BMI) is negatively correlated with GLP-1-coordinated β-cell levels, indicating a link between circulating adiposity and insulin secretion levels in humans [30]. In addition, hub cells, a special type of pacemaker β-cells, exert disproportionate control over the islet’s response to glucose. The islets are connected into a network by hub cells and follower cells to produce rhythmic activity for insulin release. The β-cell network is disrupted when hub cells are silenced, whereas calcium dynamics and insulin secretion are unaffected when follower cells are silenced. Notably, these hub cells are sensitive to pro-inflammatory and glycolipotoxic injury and lead to β-cell dysfunction [31].

The regulation of blood glucose metabolism in humans requires the involvement of both β-cells and other endocrine cells in the pancreas [32]. In 2018, Diaz et al. showed that in humans, glucagon secreted by α-cells is as important as β-cells in contributing to the glucose set point and that the action of neighboring α-cells must augment β-cell secretion to establish the human glucose set point. These findings have implications for diabetes transplantation and regenerative therapies, as restoring normal blood glucose levels may require more than just replacing β-cells. In addition, therapeutic strategies using glucagon receptor antagonists as hypoglycemic agents need to be reevaluated because they may reset the overall glucagon level in the organism [27]. Therefore, for studying MetS disease mechanisms and therapeutic strategies for diabetes, it may be more advantageous to generate intact islet-like organs in vitro than to differentiate cells into specific cell types.

### 2.2. Progress of Pancreatic Organoids

Pancreatic islet-like organs are derived from two types of stem cells: pluripotent stem cells (PSCs) and adult stem cells (ASCs) [33], and the more maturely studied organoid development strategies are mainly derived from PSCs. In 2006, Shinya Yamanaka et al. successfully reprogrammed adult fibroblasts to induced pluripotent stem cells (iPSC). Human iPSC possess pluripotent stemness like human embryonic-derived stem cells, but they do not have to be obtained from human embryos, avoiding ethical issues [34].

Pancreatic organoid development focuses on islet-like organs that generate GSIS, and pancreatic β-cells are considered the most crucial component of islet function. Previous research has focused on the generation of stem cell-derived β (SC-β) cells.

**Figure 1:**
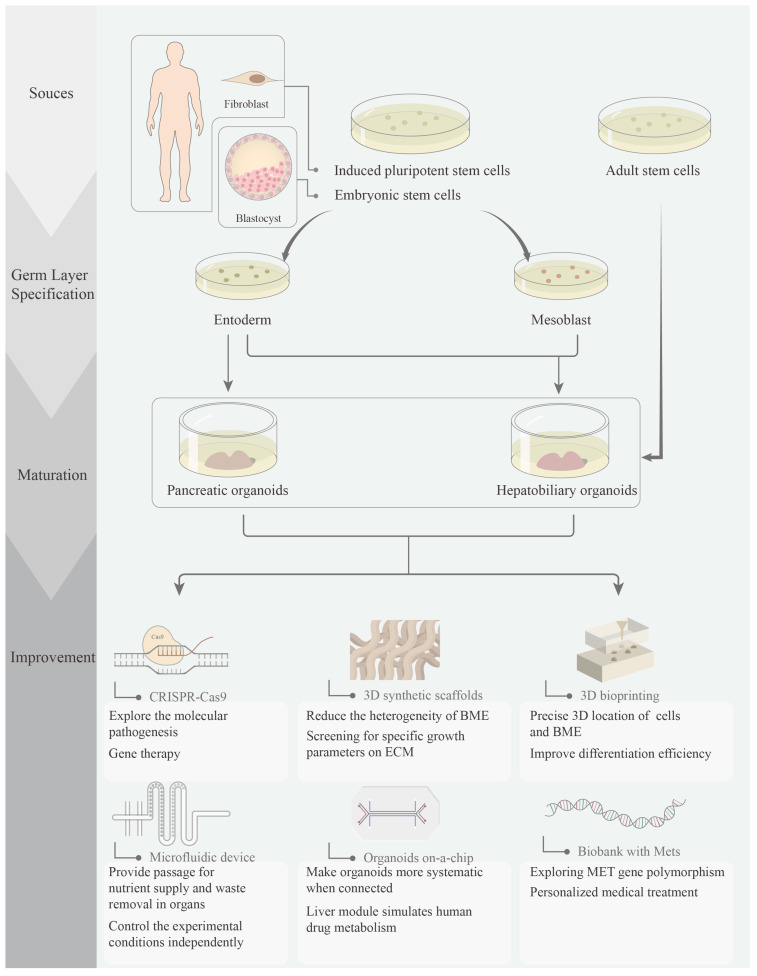
Formation and improvement of pancreatic organoids and hepatobiliary organoids. The iPSC-derived organoids are easy to retrieve and avoid ethical issues, while the ASC-derived organoids taken from MetS patients can retain the genetic characteristics of the disease, and the resulting organoids can be used for the construction of biobanks.

The basic principle of islet-like organogenesis from pluripotent stem cells is the progressive differentiation of hESCs or iPSCs through defined developmental stages, from the final endoderm, primitive intestinal duct, hind foregut, pancreatic progenitor, and endocrine progenitor to β-cells [35,36,37,38], with genetic markers identified for each cellular stage, such as PDX1 and NKX6.1, typical markers for pancreatic progenitors, and NGN3, typical endocrine precursor markers [39,40], as well as differentiation by exposing cells to various growth factors and small molecules that activate or inhibit embryonic signaling pathways in specific doses and sequences, such as lymph node activator, WNT, retinoic acid, FGF, bone morphogenetic protein (BMP), and Notch [41,42,43] (Figure 1).

However, in previous studies, immaturity of SC-β cells was demonstrated by poor glucose responsiveness, multihormonal features, and a preference of multihormonal cells for α-cells over β-cells, as cell–cell interactions play an important role in the regulation of cell fate specification and islet function [44,45,46]. Therefore, 3D culture systems for pancreatic tissue were developed, mainly including suspension 3D and scaffold 3D culture systems. The suspension 3D culture system takes advantage of intercellular self-organization. Pagliuca et al. used a stir plate to generate 100–200 um clusters of cells from hPSCs cultured in rotating flasks. Morphologically and functionally, these cell clusters resemble native human islets. Unfortunately, few non-β endocrine cells were detected compared to human islets [42]. Scaffold 3D culture systems utilize extracellular matrix (ECM) components as scaffolds to facilitate three-dimensional structure formation and cell–matrix interactions. Islet-like organs cultured in microporous scaffolds by Youngblood et al. in 2019 showed improved control of islet-like organ size and cell–cell interactions. These islet-like organs had more mature marker expression and performed better in GSIS than their counterparts in suspension culture [47]. Recent findings show that Yoshihara et al. successfully generated immune-evading human islet-like organs (HILOs) from iPSCs. HILOs overexpressing the immune checkpoint protein programmed death-ligand 1 (PD-L1) were shielded from immune destruction and could maintain cellular homeostasis in immunized mice for approximately 50 days [48].

Vascularization of islet organoids is essential for promoting GSIS, on the one hand, because the pancreas is one of the most vascularized organs, with most β-cells having at least one point of contact with the capillary bed [49]. On the other hand, contractile cells in the islets, including pericytes and smooth muscle cells, also play an important role in insulin secretion and glycemic control [50]. It has been demonstrated that activating β-cells by raising the blood glucose level inhibits pericytes, dilates islet capillaries, and boosts regional blood flow. In type 2 diabetes, pericyte coverage of islet capillaries decreases dramatically, suggesting that in diabetic conditions, islets lose the mechanism to control their own blood supply, which may lead to an insufficient release of insulin into the circulation, further worsening glycemic control [50]. In addition, the vascularization of pancreatic islet-like organs can solve the problem of insufficient nutrition and oxygen supply to deep cells in the in vitro culture of islet-like organs. The majority of islet-like organ vascularization is accomplished by co-culturing endothelial and endocrine cells. In 2018, Taniguchi’s team reported that cell lines, native tissue fragments, and iPSC spheroids co-cultured with human umbilical vein endothelial cells (HUVECs) and mesenchymal stem cells (MSCs) in Matrigel were able to form vascularized pancreatic organoids. This method is still underdeveloped for in vitro organoid formation, but clearly improves vascularization in mice after transplantation and the efficacy of diabetes [51,52]. In 2020, Palikuqi et al. transformed mature human endothelial cells into “reset” vascular endothelial cells (R-VECs), which form a perfusable and plastic vascular plexus, perfuse glucose-responsive insulin-secreting human islets, and establish an adaptive vascular ecotone that differentially regulates and adapts to organoids in a tissue-specific manner. The 3D R-VECs vascular plexus established by this type of method is self-organizing, high volume, and maintains its angiogenic potential in a wide range of serum-free medium compositions, with the characteristic that it can be cultured for long periods of time [53] (Table 1).

In general, however, the pancreatic organoids that have been grown so far are still not mature enough. The main problems are the low capacity for glucose-responsive insulin secretion, which leads to low efficiency after transplantation, and the impossibility of long-term culture, which limits further research, all of which are challenges that need to be solved for the further development of islet-like organs.

### 2.3. Pancreatic Organoids and Endogenous Regeneration of Metabolic Syndrome

There are numerous studies aimed at improving the maturation of β-cells in pancreatic organoids, whereas the lack of maturation of the produced pancreatic organoids is a significant issue in the study of islet-like organoids [45]. On the other hand, β-cell dysfunction is highly correlated with the severity of MetS, and improving β-cell dysfunction is an important strategy to improve MetS and prevent the progression of MetS to diabetes [54]. Therefore, strategies to promote further maturation of pancreatic organoids may provide new insights and ideas to improve β-cell dysfunction in MetS.

An important mechanism leading to β-cell injury in vivo under metabolic stress is the dedifferentiation of β cells. Early in 2012, Talchai’s group demonstrated that the re-emergence of endocrine progenitor-like cells in adulthood results from β-cell dysfunction [55]. For example, β-cells in T1DM [56] and T2DM [55] adapt to immune and metabolic stressors by reverting to an immature state, respectively, which partially explains the loss of β-cell population function in diabetes. Thus, adaptation to a dedifferentiated, immature cell state may be an aggressive, protective process that allows β-cells to escape immune assaults or metabolic stress-induced cell death and induces the redifferentiation of immature β-cells into mature β-cells, possibly providing a new direction for improving MetS and diabetes treatment.

Exploring whether pluripotent stem pancreatic β-progenitor cells still exist in the adult pancreas and can stimulate the redifferentiation and maturation of this adult stem cell is essential to solving this problem. Due to the similarity between in vitro organoid culture and the in vivo developmental pathway, the in vitro case of promoting islet organoid maturation may provide inspiration for finding a suitable differentiation pathway to promote the redifferentiation and maturation of damaged and dedifferentiated β-cells.

#### 2.3.1. Islet Regeneration

Strategies for endogenous islet regeneration are derived either from the mitosis of preexisting cells or from the mature differentiation of islet progenitor cells with the ability to proliferate and expand. There are molecular mechanisms that directly manipulate β-cells regarding the cell cycle that can force human β-cells to proliferate; however, genetic mutations in cell cycle genes can also lead to rare pancreatic endocrine hyperplasia, such as insulinoma in humans [57,58,59]. Therefore, the search for progenitor cell populations of β-cells in the adult pancreas could potentially promote endogenous regeneration of pancreatic islets.

Presently, the prevailing view is that the replenishment of adult pancreatic β-cells is mainly dependent on their self-replication [60], and the existence of progenitor cells that support β-cell regeneration has been a controversial topic.

Some specific cell populations are considered pancreatic progenitor or progenitor-like cells that have the ability to differentiate into insulin-secreting cells in vitro, including nestin-positive cells isolated from mouse pancreas [61], Ucn3-negative primitive β-cells from adult mice [62], P2RY1^+^/ALK3^+^ cells isolated from human pancreatic ducts [63], and high aldehyde dehydrogenase activity from human fetal and adult pancreatic cells [64]. Recently, a new population of Procr^+^ endocrine progenitor cells was found in the pancreatic islets of adult mice [65].

It is still unknown whether progenitor cells for β-cell regeneration exist in the adult pancreas, as the majority of these studies were conducted on animal or human fetal islets, or there was no genealogical tracing, making it impossible to determine whether these cells reflect the regeneration of new β-cells or dedifferentiation of existing cells.

#### 2.3.2. Oxidative Stress

In MetS, a high-energy diet can increase the metabolic load of mitochondria [66], which can form excess ROS as a byproduct, and ROS can lead to oxidative stress, which can result in cellular damage [67]. It has been found that the estrogen-related receptor (ERR) can act as a novel redox sensor and effector for ROS defense programs, and notably, increased ERRγ expression/activation is a hallmark of oxidative stress triggered by mitochondrial disruption [68]. In parallel, in a research strategy to promote islet-like organoids’ maturation, researchers have discovered that induced expression of ERR in iPSC-derived β-like cells improves maturation for function in vitro and glucose-responsive production of human insulin in vitro [69]. Recent studies have demonstrated that ERRγ maintains pancreatic alveolar cell function by regulating cellular metabolism and that ERRγ deficiency-induced mitochondrial dysfunction further triggers autophagy dysfunction, stress on the endoplasmic reticulum, and the generation of reactive oxygen species, leading to cell death [70]. Therefore, ERRγ may be a good target for restoring islet function and treating metabolic diseases.

#### 2.3.3. Circadian Rhythm

Islet cells have an internal clock that coordinates the circadian oscillations of the transcriptome. The CLOCK/BMAL1 heterodimer binds to the key islet transcription factor PDX-1 and recruits islet cell-specific enhancers. This process regulates insulin secretion. Disruption of the β-cell-specific clock also inhibits β-cell replication and promotes DNA damage-induced apoptosis, ultimately resulting in pancreatic failure [71]. Studies of insulin-like organoids have identified epigenetic and molecular circuit mechanisms of circadian rhythms and demonstrated that circadian rhythms induce the maturation of insulin-like organs and multiply the efficiency of insulin secretion. In 2020, Alvarez-Dominguez et al., in an epigenetic study investigating the fate of islet cells of stem cell origin, unexpectedly found that the activation site opened at week 3 of SC-β cell differentiation had core biological clock activators (CLOCK and ARNTL/BMAL1) in the most abundant TF binding motif. They also verified that a key function of SC-β cells, glucose responsiveness, could be improved by circadian regulation [72], possibly indicating that regular exercise in MetS patients has the potential to restore the function of damaged β-cells.

## 3. Hepatobiliary Organoids

The liver also plays an important role in regulating blood glucose and lipids. Insulin resistance in MetS leads to hepatic steatosis, and the resulting steatotoxicity can induce mitochondrial dysfunction, endoplasmic reticulum stress, and hepatocyte injury and death, progressing from non-alcoholic fatty liver (NAFL) to non-alcoholic steatohepatitis (NASH), hepatocirrhosis, and hepatocellular carcinoma (HCC) [3,73]. NAFL and NASH are uniformly referred to as “non-alcoholic fatty liver disease” (NAFLD), a representative event of MetS in the liver. NAFLD activity is defined by scoring the severity of three histologic features (steatosis, inflammation, and hepatocyte expansion), and the disease stage is defined by the fibrosis stage [74]. End-stage liver failure associated with cirrhosis due to the progressive damage of NAFLD has become the second most common reason for liver transplantation.

### 3.1. The Formation of Liver Fibrosis Requires the Cooperation of Different Cells

Recent findings suggest that long-term accumulation of hepatic fat is not strongly related to hepatitis progression but is causally linked to liver fibrosis, which is a major determinant of clinical outcome in patients with NAFLD [75,76,77,78], and that preventing the progression of liver fibrosis may be a key step in preventing the progression of MetS to cirrhosis or even liver cancer.

Hepatic fibrosis is the formation of fibrous scarring due to the accumulation of extracellular matrix (ECM) proteins, most of which are type I and type III cross-linked collagens that replace damaged normal tissue [79,80]. Its formation involves the involvement of multiple cells in the liver, mainly hepatic stellate cells (HSCs), Kupffer cells, portal fibroblasts, hepatocytes, and bile duct reactions of bile duct cells [80].

HSCs are considered the main cellular component of the cause of liver fibrosis and are located in the space of Disse. When the liver is injured by inflammation or mechanical stimulation, hepatic stellate cells are activated or transdifferentiated into myofibroblast-like cells. Myofibroblasts produce excessive amounts of synovial actin and collagen, which are involved in the formation of liver fibrosis and the reconstruction of intrahepatic structures, and, on the other hand, increase the intrahepatic sinusoidal pressure through cellular contraction [81].

Kupffer cells are the resident macrophages in the liver. They can be derived from hematopoietic stem cells and are part of the mononuclear phagocyte system. In response to liver damage, Kupffer cells release large amounts of inflammatory cytokines and chemokines, which play multiple roles in the pathogenesis of NAFLD [82].

Portal vein inflammation is associated with fibrosis progression, possibly due to the production of portal vein-proliferating myofibroblasts by portal vein fibroblasts. Activation of hepatic myofibroblasts due to chronic liver injury is an important factor in developing hepatic fibrosis and can produce fibrous scarring in liver fibrosis [83].

The ductal response (DR) is characterized by reactive bile duct hyperplasia induced by liver injury. The proliferating bile ducts mediate the proliferation and activation of stellate cells through various signaling pathways, promoting the formation of hepatic fibrosis. Bile duct reactive lesions can arise not only from preexisting bile duct cells but also from bile duct chemotaxis of hepatocytes or activated and differentiated hepatic progenitor cells. The higher the degree of hepatic fibrosis, the higher the DR score in NASH patients [84].

In summary, the above cellular components are essential if liver-like organ models are to mimic NAFLD disease progression, liver fibrosis outcomes, and treatment strategies.

### 3.2. Progress of Hepatobiliary Organoids

#### 3.2.1. Hepatobiliary Organoids

The liver is an important organ with multiple functions, including detoxification, digestion, and metabolism, provided by hepatic parenchymal and nonparenchymal hepatocytes organized into functional units called lobules. Although the liver comprises many different types of cells, hepatocytes and biliary epithelial cells make up the majority of them. The bile ducts, which run throughout the liver, play an important role in both the metabolic functions and the fibrotic process of the liver; therefore, this paper focuses on the hepatobiliary organoids, which, like the pancreatic organoids, have both PSC and ASC sources.

ASC-derived hepatobiliary organoids are predominantly derived from epithelial cells. In mice, the WNT pathway has emerged as a major driver of epithelial ASC. In epithelial cells, Lgr5 marks the majority of active ASC. Injury-induced Lgr5^+^ cells, after isolation from tissues, can be clonally expanded into organoids in an environment that mimics a stem cell ecotone, consisting mainly of the early bile duct and hepatocyte markers. The human adult liver organoid is derived from liver tissue [85]. Human adult liver-like organs are derived from EpCAM^+^ bile duct epithelial cells within liver tissue and maintain genomic stability after long-term cultivation [86].

PSC-based liver-like organoids are built using a developmental process in such a way that the development of the liver begins with an endocytic mass, which then gradually develops into an embryo, and the hind foregut of the endoderm develops into the liver when the triple germ layer is formed. The liver is formed by the growth of the ventral foregut epithelium, which first develops into the hepatic bud structure. The liver buds give rise to hepatocytes and bile duct epithelial cells, while the adjacent mesenchyme comprises hepatic fibroblasts and hepatic stellate cells [7]. Therefore, the development of hepatobiliary organoids requires the combined action of endoderm and mesoderm. A portion of PSC cells is first induced to generate endodermal cells, and then another portion is induced to become mesodermal cells, which self-organize into hepatobiliary organoids under the combined action of both stem cell types. This approach originated in 2013, when Takebe et al. used cross-signaling between endodermal epithelial cells, mesenchymal cells, and endothelial progenitor cells to generate human liver bud-like tissue. In 2017, Takebe’s group further advanced liver organoid development so that both endoderm and mesoderm were derived exclusively from multifunctional induced stem cells (iPSCs) [87,88] In 2019, Wu et al. induced both endodermal and mesodermal differentiation to outline key aspects of early hepatogenesis in a parallel manner, more in line with the developmental sequence in vivo, and demonstrated the role of cholesterol and the herbal small molecule MIX in promoting hepatobiliary organoid maturation [89]. In the same year, Ouchi et al. developed a method to generate a multi-tissue liver organoid via PSC that includes hepatic stromal subtype cells in addition to hepatobiliary cells. The presence of hepatocytes, cholangiocytes, hepatic stellate cells, Kupffer cells, and parthenogenetic hepatic progenitor cells with dual differentiation abilities of hepatocyte and cholangiocyte lineages was confirmed using single-cell RNA sequencing (SCRNA-seq) [90]. In 2020, Ramli et al. reported the generation of PSC-derived hepatic epithelial-like organs containing functionally interconnected hepatic and biliary compartments, and their study was the first to show that hepatocytes and biliary cells can be generated from hepatoblast cells and can generate organoids of consistent shape, size, and structure with high throughput. Furthermore, the method generates liver-like organs in the absence of Matrigel, allowing easy retrieval of analogs for downstream applications, but unfortunately, other cell types, such as Kupffer cells and hepatic stellate cells, are missing from this liver-like organelle [91]. In the same year, Shinozawa et al. reported the generation of iPSC-derived hepatobiliary organoids. They used a high-speed real-time imaging platform for high-throughput drug screening and analysis of multiple readouts of interactions between other factors, such as mitochondrial stress [92].

The differentiation approach also mimics the progenitor ecological niche, where the organoid is exposed to various growth factors and small molecules that activate or inhibit embryonic signaling pathways in specific doses and sequences.

#### 3.2.2. NAFLD Modelling

Although rodent models have played an important role in the study of liver development and NAFLD disease mechanisms, as mentioned earlier, animals and humans still differ in many ways. Little overlap was found between the two at the genetic level when comparing the liver gene expression profiles of different NASH mouse models and NASH patients [93].

In 2018, Lyall et al. developed a NAFLD model of hepatocyte-like cells (HLCs) derived from human embryonic stem cells, where exposure to lactate, pyruvate, and octanoic acid induced a NAFLD phenotype in HLCs with a transcriptional and metabolomic dysregulation consistent with that present in human NAFLD [94]. Subsequently, Sinton et al. demonstrated that this model possesses transcriptional and metabolic features associated with human hepatic steatosis and that this model of hepatic steatosis is replicable, scalable, and highly simulates transcriptomic, epigenomic, metabolomic, and proteomic effects in real humans [95]. This model demonstrates that PSC-derived models can replace animal models and primary hepatocytes for the study of NAFLD but cannot be called “organoid” due to their single-cell nature. In contrast, hepatobiliary organoids developed in recent years have proven to be superior models of NAFLD due to their multicellular nature. For example, the hepatobiliary organoid developed by Ouchi in 2019 reproduced the main features of steatohepatitis, including steatosis, inflammation, and fibrosis phenotypes, after free fatty acid (FFA) treatment and reflected the severity of fibrosis [90]. In 2020, the gene expression profile of the organoid developed by Ramli’s group that was co-incubated with FFA was similar to that of liver tissue from NASH patients and led to structural changes associated with NAFLD, such as attenuation of the bile duct network and ductal response [91]. In 2021, the McCarron group derived and differentiated bifunctional conduit-like organs from stem cells isolated from the irreversibly damaged livers of NASH patients. The transcriptome of organoids derived from NASH livers but not healthy livers showed significant upregulation of pro-inflammatory and cytochrome P450-related pathways as well as known liver fibrosis and tumor markers, with the degree of upregulation dependent on patient specificity. Functionally, NASH liver-like organs exhibit reduced transmission/growth capacity and characteristics of NASH livers, including reduced albumin production, increased free fatty acid-induced lipid accumulation, increased sensitivity to apoptotic stimuli, and increased cytochrome P450 metabolism. After hepatic differentiation, NASH liver organoids exhibit a reduced ability to dedifferentiate back to a biliary state, which is consistent with the known reduced regenerative capacity of NASH livers [96].

The study of NAFLD hepatobiliary organoids provides a new approach, thus facilitating the discovery of effective treatments, while the exploitation of liver organoids directly from NAFLD patients with irreversibly damaged livers opens new experimental avenues for personalized disease modeling and drug development (Table 2).

### 3.3. Hepatobiliary Organoids and Endogenous Regeneration of Metabolic Syndrome

NASH and liver fibrosis can be reversed when harmful substances are removed from the liver, which has a high regenerative capacity. Interventions in the early stages of NAFLD, such as a healthy diet, regular exercise, and moderate exercise, have a good chance of reversing NASH and liver fibrosis [97]. Although there are no FDA-approved effective anti-fibrotic drugs, studies have demonstrated the efficacy of the peroxisome proliferator-activated receptor γ (PPARγ) agonist pioglitazone, vitamin E [98], and the cognate bile acid receptor (FXR) agonist obeticholic acid [99]. Clinical trials have confirmed improvements in both disease activity and fibrosis in patients. Unfortunately, trials using direct anti-fibrotic agents (e.g., simtuzumab) to inhibit fibrosis have been unsuccessful [100], and fibrosis regression in advanced fibrosis and cirrhosis remains a challenge. Patients with advanced cirrhosis have severe fibrosis and diminished liver regeneration, and liver transplantation has many problems that are difficult to overcome, mainly donor shortage and immune rejection [101].

#### 3.3.1. Liver Regeneration

The regenerative capacity of the adult liver is amazing. Under normal conditions, less than 2% of hepatocytes and bile duct cells proliferate. However, after injury, the liver demonstrates a strong proliferative response to regeneration and can rapidly re-generate within a few days in many species as long as more than 2/3 of the liver is not removed [102,103]. The regenerative mechanism of the liver is primarily mitotic; therefore, regeneration after partial hepatectomy is characterized by typical phenotypic fidelity [102].

However, when one of the hepatocytes or biliary cells loses the ability to regenerate, their alternative regeneration scheme as reciprocal stem cells is activated, and progenitor cells with hepatobiliary properties originate from biliary cells and gradually transform into hepatocytes in the case of liver regeneration where hepatocyte proliferation is inhibited; similarly, in the case of inhibited biliary cell proliferation, periportal hepatocytes transform into biliary cells in situ, mimicking a similar transformation that occurs during embryonic development [104].

A team of researchers has observed dual-phenotype cells in human chronic liver disease, suggesting a cell identity switch between hepatocytes and bile duct cells or parthenogenetic liver progenitor cells (LPCs). This observation suggests another purpose for injury-induced cellular plasticity: An escape mechanism that allows cells to retain regenerative capacity after injury. This hypothesis is supported by a chimeric lineage tracing model that redifferentiates back to hepatocytes after injury cessation [105].

In 2013, Hattoum et al. discovered that in fulminant liver failure, a large number of proliferating EpCAM^+^ bile duct epithelial cells could be observed when 80% of hepatocytes were lost [106]. Subsequently, Huch’s group found that EpCAM^+^ bile duct epithelial cells could be easily grown in vitro as bipotent stem cells into 3D-like organoids [86].

This year, Gao’s group identified a new biphenotypic EpCAM^+^Gli1^+^ cell population located in peribiliary and periportal pericyte ecological niches that facilitates hepatocyte regeneration during chronic liver injury. Genetic lineage tracing using a dual recombinase showed that the Gli1^+^ non-hepatocyte population could give rise to hepatocytes after chronic liver injury. EpCAM^+^Gli1^+^ cells have a greater ability to form functional hepatocytes as organoids in vitro and exhibit greater liver regeneration when transplanted into FRG mice in vivo [107].

Overall, these findings suggest that there are parthenogenic progenitor cells in the liver that can serve as a new source of liver progenitor cells that not only contribute to liver repair and regeneration but are also a major source of cells for the cultivation of ASC-derived hepatobiliary organoids. But can these liver progenitor cells, which can be used to cultivate hepatobiliary organoids, use their regenerative capacity to promote endogenous regeneration of the MetS and the regression of liver fibrosis? So far, these issues have become hot topics in related fields, such as the in vitro injection of mesenchymal hepatocytes (MSCs), macrophages, and exosomes secreted by MSCs, all of which have been shown to promote the regeneration of progenitor cells in the liver and the regression of liver fibrosis in MetS [108], while the hepatobiliary organoids provide a more superior model for such studies.

#### 3.3.2. Regression of Liver Fibrosis

The regression of liver fibrosis is associated with a decrease in the production of pro-inflammatory or fibrogenic cytokines, the disappearance of hepatic myofibroblasts, the inhibition of ECM production, an increase in collagenolytic activity, and the dissolution of fibrous scarring [97]. The shutdown of the inflammatory response is key to liver regeneration after acute liver injury (ALI) and fibrosis reconstruction after chronic injury. In addition, phagocytosis is a key process in the elimination of any inflammatory response, and in the case of liver damage, macrophages are phagocytes specifically responsible for the removal of large numbers of dead cells from the liver [109].

The study of hepatic macrophages has shown increasing importance in recent years, including not only liver-resident macrophages, that is, Kupffer cells, but also macrophages derived from monocytes. Macrophages can support biliary tract regeneration, promote fibrotic remodeling by inhibiting hepatic stellate cell activation, and promote liver regeneration by removing dead cells [82,110]. Tsuchiya’s group analyzed the therapeutic potential of small extracellular vesicles (sEVs) derived from interferon-γ (IFN-γ)-pretreated MSCs (γ-sEVs). In vitro, γ-sEVs were effective in inducing the aggregation of anti-inflammatory macrophages with high motility and phagocytic capacity in damaged areas, and γ-sEVs were more effective than sEVs in ameliorating inflammation in a mouse model of cirrhosis [111]. Although the study confirmed the anti-inflammatory and regenerative potential of MSCs and macrophages in vivo, MSC-γ- sEVs did not directly reduce the activation of HSCs, which are the main cells involved in the progression of liver fibrosis. Their apoptosis or transformation is also a key factor in the regression of liver fibrosis. The study was conducted mainly in mice, and whether it can be extended to humans still needs to be explored. Hepatobiliary organoids can provide a complementary study in this regard.

For example, Kimura et al. designed human organoid combinations for steatohepatitis from a multi-donor human progenitor cell bank and found that the GCKR-rs1260326-T allele increased disease severity only in the diabetic state but prevented fibrosis in the non-diabetic state [112].

## 4. Perspectives of Metabolic Syndrome-Related Organoids

Organoids are spontaneously formed multicellular structures that provide a reliable model for studying early development and certain diseases. MetS is a systemic disease that affects multiple organs and tissues throughout the human body. A single organoid is not a good model for studying metabolic syndrome, as it lacks the organ-to-organ and system-to-system interactions necessary to study the disease. Secondly, the current immaturity of organoids and the inability to produce them on a large scale and in a standardized manner have created significant limitations for the study of various diseases, especially systemic diseases such as Mets. However, the combination of organoids with other technologies is expected to break the metabolic syndrome research bottleneck. Some of the latest research results on engineered organoids are presented below (Figure 1).

### 4.1. CRISPR-Based Gene Editing

Compared to animal models and primary cell models, PSC-derived organoids can be altered by CRISPR-Based, an efficient gene editing technology, to alter the expression of a gene in organoids. This method can help to improve the efficiency or maturity of organoid construction and can also be used to explore the impact of the gene on the whole process of disease development, providing strong support for the molecular pathogenesis of related diseases and the subsequent development of gene therapy.

For example, l’Hortet et al. used this technique to confirm the important role of the SIRT1 gene in human fatty liver formation, found that increased fatty acid biosynthesis exacerbated fat accumulation by differentiating edited iPSCs into hepatocytes and knocking out SIRT1, and established a human fatty liver model with human SIRT1 knockout iPSC-derived hepatocytes that obtained a pro-inflammatory phenotype and shared a similar lipid and metabolic profile to the human fatty liver [113]. Just recently, Hendriks’ group used this technique to knock out the APOB and MTTP genes in human fetal hepatocyte-derived organoids, deletions of which are responsible for two monogenic lipid disorders predisposing to NAFLD: familial hypolipoproteinemia and abetalipoproteinemia. APOB^−/−^ and MTTP^−/−^ mutant organoids constitute a natural steatosis organ model and can be maintained in long-term culture at levels that maintain a stable level of steatosis. The group used the lipotropic organoids to build a CRISPR-Based screening platform, through which FADS2 was found to be a key regulator of lipotrophy. While FADS2 deficiency exacerbated the steatosis phenotype, overexpression of FADS2 resulted in reduced steatosis [114].

### 4.2. 3D Synthetic Scaffolds

Currently, effective expansion of organoids requires matrix or basement membrane extraction (BME). However, most organoid cultures use Matrigel as BME. Matrigel is derived from mouse sarcomas, and its composition is heterogeneous and varies significantly from batch to batch, making it impossible to standardize organoid models for large-scale culture and the reproduction of results more difficult [115]. Compared to animal-derived matrices, protein- or polysaccharide-based biopolymers can be recombinantly produced with reduced variability. In addition, synthetic matrices offer the opportunity to experimentally isolate the stiffness, bioactivity, and variability of the environment in which the organoid grows, allowing screening methods to be developed to investigate the impact of each parameter on stem cell fate [116,117,118].

For example, enrichment of certain ECM components, such as laminin, promotes the conversion of bipotential pancreatic progenitor cells to endocrine cell specification, whereas exposure to other ECM components induces ductal cell differentiation, implying that stage-specific scaffolds may promote endocrine differentiation in vitro and improve induction efficiency [119].

### 4.3. 3D Bioprinting

Bioprinting is a promising and innovative biomanufacturing strategy for precisely locating biological agents, including living cells and extracellular matrix (ECM) components, in defined 3D layered tissues to create artificial multicellular tissues/organs [120]. An engineering approach using bioprinting to control initial cell density, size, and shape of cell aggregates, cell–ECM interactions, and biochemical gradients will provide more precise guidance for the generation of PSC-derived organoids [121].

It has been demonstrated that the effectiveness of differentiating hPSCs into SC-β cells is closely related to cell density, cell line, and induction protocol [122,123]. In 2018, Memon et al. were able to improve the induction efficiency of PDX1^+^/NKX6.1^+^ pancreatic progenitor cell populations by manipulating the replating density [124]. In 2019, Bernal et al. demonstrated that volumetric bioprinting via optical tomography could shape gelatin hydrogels containing organoids into complex centimeter-scale 3D structures in less than 20 s [125]. Last year, Daly et al. demonstrated a bioprinting method that transfers high-resolution spheroids into homogeneous supporting hydrogels, allowing them to be patterned and fused into high-cell-density microtissues with defined spatial organization [126].

### 4.4. Organoids in a Microfluidic Device

The problem of inaccessibility during organoid culture has been a major problem for researchers, and the usual solution is to periodically disassemble and reseed the organoids onto the culture medium, which makes them unsuitable for long-term research observations. Microfluidic devices are a promising tool for integrating channels for nutrient supply and waste removal within organoids and enabling autonomous control of experimental conditions.

In 2020, Liu et al. developed a droplet microfluidic system for the regulated fabrication of hybrid hydrogel capsules, which permits large-scale 3D culture and the formation of functional and uniform islet-like organoids derived from hiPSC. The produced hybrid capsules exhibit high homogeneity and are stable, biocompatible, and infiltrative [127].

### 4.5. Organoids on a Chip

In 2018, Koike’s team developed a scheme for the sequential construction of liver, biliary, and pancreatic (HBP) structures from three-dimensionally cultured human pluripotent stem cells (PSCs). Unfortunately, such a scheme cannot yet be clearly discernible spatially and requires further development of maturation [128], which, in combination with organoid microarray technology, may be used to model connectivity between organoids from other stem cell sources [129].

In 2022, Tingting Tao’s team designed a liver and islet-like organ co-culture system on a chip capable of studying organ–organ interactions under perfusion co-culture conditions for up to 30 days. The system provides a powerful method to study the feedback loop within the human liver–pancreatic islet axis that maintains glucose levels in the normoglycaemic range in vitro, a result that cannot be achieved in single organ culture; both liver and islet organoids exhibit mitochondrial dysfunction and reduced glucose transport capacity under hyperglycaemic conditions, which can be alleviated by metformin treatment, suggesting that the liver-islet organoids system is able to mimic the key pathological features of T2DM. A distinct advantage of this system is its ability to mimic the human-relevant functional coupling of liver and islet organs in response to external hyperglycemic stimuli and drugs, which is not easily studied in monolayer cell cultures or animal models [130].

### 4.6. Biobanks and MetS

MetS and genetic susceptibility are closely related, and genetic polymorphisms play an important role in MetS [131,132]. Extracted ASC-derived organ tissues from MetS patients can preserve the inherited characteristics of the disease, and the resulting organ tissues can be used for the construction of biobanks. This will facilitate global MetS research and personalized treatment, such as the development of appropriate diet, exercise, and medication plans for a particular nucleotide polymorphism. As early as 2008, genome-wide association scans identified PNPLA3 (rs738409[G], encoding I148M) to be closely associated with increased levels of liver fat and liver inflammation [133]. In a newly published paper by Hendriks’ group, demonstrating that genetic susceptibility to NAFLD affects the efficacy of relevant drugs, they found that carrying the PNPLA3 I148M variant attenuates organ response to fatty liver drugs, which is particularly evident in the FXR–FGF19 drug axis, and this study provides evidence for possible future personalized medicine for NAFLD [114].

The establishment of such biobanks has been the subject of global efforts. Zeng et al. created isogenic human ESCs (hESCs) with mutations in type 2 diabetes susceptibility genes identified by genome-wide association studies (GWAS). In pancreatic β-like cells derived from these cell lines, CDKAL1, KCNQ1, and KCNJ11 mutations were found to cause impaired glucose secretion in vitro and in vivo, consistent with defective glucose homeostasis [128]. Recently, Kimura et al. designed combinations of human organoids for steatohepatitis from a multi-donor human progenitor cell bank to investigate the effect of metabolic status on genotype–phenotype associations. Precision hepatology is supported by a comprehensive arsenal of mechanistic, discriminative, and therapeutic reasoning [112].

## 5. Conclusions and Perspectives

MetS has gradually become a focus of medical research due to its widespread prevalence in populations worldwide and the serious dangers of its derivatives, diabetes, cirrhosis, and cardiovascular disease [1,2,4]. Due to the complexity of the disease itself and the limitations of research models, effective external interventions and treatments for this group of diseases have not yet been developed.

Since the invention of iPSC and the discovery of various in situ stem cells in vivo, organoid research has flourished and has many advantages that primary cell culture and animal models cannot reach. However, due to the limitations of current technology, organoids continue to have numerous, insurmountable limitations that prevent them from completely replacing other models. These shortcomings include, but are not limited to, the following: (1) insufficient maturity of the resulting organoid, which is approximately equivalent to the human fetal level but still differs significantly from the adult phenotype; (2) insufficient complexity of organoid construction, primarily incomplete cell types, such as the lack of immune cells that are important in disease progression; (3) difficulty controlling multicellular co-culture conditions, which prevents large-scale standardized production; (4) contradiction between organoid volume and nutrient supply, which prevents long-term cultivation; and (5) the ability to introduce spontaneous mutations at a relatively high rate compared to primary cell culture [134]. However, it is believed that as organoids are combined with other engineering-based technologies, they are expected to become superior to other models of existence, especially for complex and comprehensive diseases like MetS.

However, it is also important to recognize that more complex and more complete organoids are not superior and that both simple and complex organoid systems have their advantages and disadvantages. The cultivation and production of simple organoids are simpler and more resource efficient, but they lack fidelity for complex diseases. Complex organoids are closer to the actual level of the human body but are difficult and labor-intensive to cultivate. Consequently, it is essential to use the most appropriate level of complexity in different studies; however, there are still no standardized guidelines for these issues.

Returning to the question posed at the beginning, can endogenous regeneration be an effective means of preventing the progression of hepatobiliary and pancreatic MetS to end-stage? The authors believe this is a promising strategy based on previous research on organoid disease models. In terms of pancreatic regeneration, many studies have demonstrated that the dysfunction of β-cells in the MetS is due to their dedifferentiation, and coupled with the fact that many studies have demonstrated that stem cells may still exist in situ in the pancreas, the strategies used in pancreatic organoid studies to promote pancreatic differentiation and maturation may also be used to promote β-cell regeneration in the MetS. In terms of liver regeneration, the presence of EpCAM^+^ bile duct epithelial cells in the liver as bipotent stem cells and strategies to promote endogenous liver regeneration by promoting the regression of liver fibrosis with in vitro injections of mesenchymal hepatocytes (MSCs), macrophages, and exosomes secreted by MSCs are also hot topics of current research. However, it has to be acknowledged that there are still major difficulties in this field. In terms of pancreatic regeneration, the main issue is that the presence of β-progenitor cells in the adult pancreas is not yet conclusive, and the target signaling pathways that drive the redifferentiated β-progenitor cells in MetS and diabetes to develop and mature again have not been identified. Due to the high regenerative potential of the liver itself, the promotion of fibrous regression and liver regeneration has become a focal point for relevant drug and regeneration research. However, some major issues remain: Valid markers or methods to enhance fibrogenesis assessment during regeneration have not yet been identified, and the mechanisms underlying the inactivation of myofibroblasts derived from HSCs or portal fibroblasts, particularly the epigenetic changes that shape the phenotype, remain to be determined. These unresolved challenges provide directions for future research.

## Figures and Tables

**Table 1:** Comparison of different pancreatic organoids.

Initial Cells	Advantages	Disadvantages	Year
hPSCs	The first strategy for producing functional cells from hPSCs developed a 6-step process by including predetermined components.	Insufficient maturity; GSIS is not high enough. Not suitable for long-term cultivation.	2014 [42]
hPSCs	It was demonstrated that endocrine cell clustering is a crucial stage in the development of hPSC-derived cells in culture.	Insufficient maturity; GSIS is not high enough. Not suitable for long-term cultivation.	2019 [43]
hPSCs	Improved control over islet organoid size and cell–cell interactions was demonstrated in islet organoids cultivated in a microporous scaffold.	Insufficient maturity; GSIS is not high enough. Not suitable for long-term cultivation.	2019 [47]
iPSCs	In vitro, WNT4 enhanced GSIS and markedly boosted mitochondrial content and oxidative metabolism. Ex vivo interferon stimulation led to reduced T cell activation and graft rejection as well as endogenous PD-L1 expression.	Insufficient maturity.	2020 [48]
iPSC, HUVECs, hMSCs	Compared to non-vascularized islets, the gene expression patterns of vascularized islet organoids more closely resemble those of native islets.	Insufficient maturity, GSIS is not high enough.	2018 [51,52]

Note: GSIS: glucose-responsive insulin secretion; hPSCs: human pluripotent stem cells; HUVECs: human umbilical vein endothelial cells; iPSCs: induced pluripotent stem cells; MSCs: mesenchymal stem cells; PD-L1: programmed death-ligand 1.

**Table 2:** Comparison of different hepatobiliary organoids.

Initial Cells	Advantages	Disadvantages	Year
Adult human EpCAM^+^ ductal cells	Demonstrate how primary human bile duct cells can be easily differentiated into 3D organoids in vitro using bipotent stem cells and how long-term cultured cells can maintain their genetic integrity.	Simple function and simple cell composition.	2015 [86]
Ductal cells derived from NASH patients’ liver	In contrast to organoids made from healthy sources, it accurately captures the pathological traits of NASH.	Only for the NASH study.	2015 [96]
hiPSCs	Improve the repeatability and scalability of organoids and provide a fully hiPSC-based platform for the generation of organ buds.	Simple function and simple cell composition.	2017 [88]
hiPSCs	IPSCs should be encouraged to co-differentiate into the hepatic, biliary, and mesodermal lineages.	Lack of HSCs and Kupffer cells. Insufficient maturity.	2019 [89]
hiPSCs	Create a repeatable procedure to produce multicellular human liver organoids with hepatocyte, stellate, and Kupffer-like cell types. Organoids that had received FFA therapy replicated important aspects of steatohepatitis, including steatosis, inflammation, and fibrosis phenotypes.	Its functional activity remained undetermined. The inter- or intra-batch organoid variability affects the sHLO phenotype such as fibrosis.	2019 [89]
hiPSCs	The use of patient-specific iPSC, the foregut stage’s storage capability, assay throughput, and multiplexed readouts for examining how other parameters, such as mitochondrial stress, interact.	Insufficient maturity. Lack of adaptive immune components.	2020 [92]
hiPSCs, HUVECs, hMSCs	The initial study demonstrated that PSC-derived human organoids could be vascularized and functional.	Simple function and simple cell composition.	2013 [87]
hPSCs	The first to demonstrate that hepatic and biliary cells may develop from hepatoblast cells and the first to produce organoids at a high rate that are uniform in terms of size, shape, and composition.	Lack of HSCs and Kupffer cells. Insufficient maturity.	2020 [91]

Note: FFA: free fatty acids; hiPSCs: human induced pluripotent stem cells; HLO: human liver organoid; HUVECs: human umbilical vein endothelial cells; hMSCs: human mesenchymal stem cells; NASH: non-alcoholic steatohepatitis.

## References

[1] Eckel R.H., Grundy S.M., Zimmet P.Z. (2005). The metabolic syndrome. Lancet.

[2] Després J.P., Lemieux I. (2006). Abdominal obesity and metabolic syndrome. Nature.

[3] Bozkurt B., Aguilar D., Deswal A., Dunbar S.B., Francis G.S., Horwich T., Jessup M., Kosiborod M., Pritchett A.M., Ramasubbu K. (2016). Contributory Risk and Management of Comorbidities of Hypertension, Obesity, Diabetes Mellitus, Hyperlipidemia, and Metabolic Syndrome in Chronic Heart Failure: A Scientific Statement from the American Heart Association. Circulation.

[4] Bence K.K., Birnbaum M.J. (2021). Metabolic drivers of non-alcoholic fatty liver disease. Mol. Metab..

[5] Xu H., Li X., Adams H., Kubena K., Guo S. (2018). Etiology of Metabolic Syndrome and Dietary Intervention. Int. J. Mol. Sci..

[6] Jain R., Lammert E. (2009). Cell-cell interactions in the endocrine pancreas. Diabetes Obes. Metab..

[7] Lancaster M.A., Knoblich J.A. (2014). Organogenesis in a dish: Modeling development and disease using organoid technologies. Science.

[8] Cabrera O., Berman D.M., Kenyon N.S., Ricordi C., Berggren P.O., Caicedo A. (2006). The unique cytoarchitecture of human pancreatic islets has implications for islet cell function. Proc. Natl. Acad. Sci. USA.

[9] Brenot F., Herve P., Petitpretz P., Parent F., Duroux P., Simonneau G. (1993). Primary pulmonary hypertension and fenfluramine use. Br. Heart J..

[10] Connolly H.M., Crary J.L., McGoon M.D., Hensrud D.D., Edwards B.S., Edwards W.D., Schaff H.V. (1997). Valvular heart disease associated with fenfluramine-phentermine. N. Engl. J. Med..

[11] Kim J., Koo B.K., Knoblich J.A. (2020). Human organoids: Model systems for human biology and medicine. Nat. Rev. Mol. Cell Biol..

[12] Clevers H. (2016). Modeling Development and Disease with Organoids. Cell.

[13] Bhaduri A., Andrews M.G., Kriegstein A.R., Nowakowski T.J. (2020). Are Organoids Ready for Prime Time?. Cell Stem Cell.

[14] Hofer M., Lutolf M.P. (2021). Engineering organoids. Nat. Rev. Mater..

[15] Castro-Barquero S., Ruiz-León A.M., Sierra-Pérez M., Estruch R., Casas R. (2020). Dietary Strategies for Metabolic Syndrome: A Comprehensive Review. Nutrients.

[16] Wilkinson M.J., Manoogian E.N.C., Zadourian A., Lo H., Fakhouri S., Shoghi A., Wang X., Fleischer J.G., Navlakha S., Panda S. (2020). Ten-Hour Time-Restricted Eating Reduces Weight, Blood Pressure, and Atherogenic Lipids in Patients with Metabolic Syndrome. Cell Metab..

[17] Wewege M.A., Thom J.M., Rye K.A., Parmenter B.J. (2018). Aerobic, resistance or combined training: A systematic review and meta-analysis of exercise to reduce cardiovascular risk in adults with metabolic syndrome. Atherosclerosis.

[18] Aminian A., Al-Kurd A., Wilson R., Bena J., Fayazzadeh H., Singh T., Albaugh V.L., Shariff F.U., Rodriguez N.A., Jin J. (2021). Association of Bariatric Surgery with Major Adverse Liver and Cardiovascular Outcomes in Patients with Biopsy-Proven Nonalcoholic Steatohepatitis. JAMA.

[19] El-Assaad W., Buteau J., Peyot M.L., Nolan C., Roduit R., Hardy S., Joly E., Dbaibo G., Rosenberg L., Prentki M. (2003). Saturated fatty acids synergize with elevated glucose to cause pancreatic beta-cell death. Endocrinology.

[20] Butler A.E., Janson J., Bonner-Weir S., Ritzel R., Rizza R.A., Butler P.C. (2003). Beta-cell deficit and increased beta-cell apoptosis in humans with type 2 diabetes. Diabetes.

[21] Atkinson M.A., Campbell-Thompson M., Kusmartseva I., Kaestner K.H. (2020). Organisation of the human pancreas in health and in diabetes. Diabetologia.

[22] Roscioni S.S., Migliorini A., Gegg M., Lickert H. (2016). Impact of islet architecture on β-cell heterogeneity, plasticity and function. Nat. Rev. Endocrinol..

[23] Ramond C., Glaser N., Berthault C., Ameri J., Kirkegaard J.S., Hansson M., Honoré C., Semb H., Scharfmann R. (2017). Reconstructing human pancreatic differentiation by mapping specific cell populations during development. eLife.

[24] Ramond C., Beydag-Tasöz B.S., Azad A., van de Bunt M., Petersen M.B.K., Beer N.L., Glaser N., Berthault C., Gloyn A.L., Hansson M. (2018). Understanding human fetal pancreas development using subpopulation sorting, RNA sequencing and single-cell profiling. Development.

[25] Nair G., Hebrok M. (2015). Islet formation in mice and men: Lessons for the generation of functional insulin-producing β-cells from human pluripotent stem cells. Curr. Opin. Genet. Dev..

[26] Capozzi M.E., Svendsen B., Encisco S.E., Lewandowski S.L., Martin M.D., Lin H., Jaffe J.L., Coch R.W., Haldeman J.M., MacDonald P.E. (2019). β Cell tone is defined by proglucagon peptides through cAMP signaling. JCI Insight.

[27] Rodriguez-Diaz R., Molano R.D., Weitz J.R., Abdulreda M.H., Berman D.M., Leibiger B., Leibiger I.B., Kenyon N.S., Ricordi C., Pileggi A. (2018). Paracrine Interactions within the Pancreatic Islet Determine the Glycemic Set Point. Cell Metab..

[28] Benninger R.K.P., Kravets V. (2022). The physiological role of β-cell heterogeneity in pancreatic islet function. Nat. Rev. Endocrinol..

[29] Dorrell C., Schug J., Canaday P.S., Russ H.A., Tarlow B.D., Grompe M.T., Horton T., Hebrok M., Streeter P.R., Kaestner K.H. (2016). Human islets contain four distinct subtypes of β cells. Nat. Commun..

[30] Hodson D.J., Mitchell R.K., Bellomo E.A., Sun G., Vinet L., Meda P., Li D., Li W.H., Bugliani M., Marchetti P. (2013). Lipotoxicity disrupts incretin-regulated human β cell connectivity. J. Clin. Investig..

[31] Johnston N.R., Mitchell R.K., Haythorne E., Pessoa M.P., Semplici F., Ferrer J., Piemonti L., Marchetti P., Bugliani M., Bosco D. (2016). Beta Cell Hubs Dictate Pancreatic Islet Responses to Glucose. Cell Metab..

[32] Jo J., Choi M.Y., Koh D.S. (2009). Beneficial effects of intercellular interactions between pancreatic islet cells in blood glucose regulation. J. Theor. Biol..

[33] Huch M., Bonfanti P., Boj S.F., Sato T., Loomans C.J., van de Wetering M., Sojoodi M., Li V.S., Schuijers J., Gracanin A. (2013). Unlimited in vitro expansion of adult bi-potent pancreas progenitors through the Lgr5/R-spondin axis. EMBO J..

[34] Takahashi K., Yamanaka S. (2006). Induction of pluripotent stem cells from mouse embryonic and adult fibroblast cultures by defined factors. Cell.

[35] Jennings R.E., Berry A.A., Strutt J.P., Gerrard D.T., Hanley N.A. (2015). Human pancreas development. Development.

[36] Petersen M.B.K., Gonçalves C.A.C., Kim Y.H., Grapin-Botton A. (2018). Recapitulating and Deciphering Human Pancreas Development From Human Pluripotent Stem Cells in a Dish. Curr. Top. Dev. Biol..

[37] Slack J.M. (1995). Developmental biology of the pancreas. Development.

[38] Polak M., Bouchareb-Banaei L., Scharfmann R., Czernichow P. (2000). Early pattern of differentiation in the human pancreas. Diabetes.

[39] Rezania A., Bruin J.E., Xu J., Narayan K., Fox J.K., O’Neil J.J., Kieffer T.J. (2013). Enrichment of human embryonic stem cell-derived NKX6.1-expressing pancreatic progenitor cells accelerates the maturation of insulin-secreting cells in vivo. Stem Cells.

[40] Kelly O.G., Chan M.Y., Martinson L.A., Kadoya K., Ostertag T.M., Ross K.G., Richardson M., Carpenter M.K., D’Amour K.A., Kroon E. (2011). Cell-surface markers for the isolation of pancreatic cell types derived from human embryonic stem cells. Nat. Biotechnol..

[41] Jennings R.E., Berry A.A., Gerrard D.T., Wearne S.J., Strutt J., Withey S., Chhatriwala M., Piper Hanley K., Vallier L., Bobola N. (2017). Laser Capture and Deep Sequencing Reveals the Transcriptomic Programmes Regulating the Onset of Pancreas and Liver Differentiation in Human Embryos. Stem Cell Rep..

[42] Pagliuca F.W., Millman J.R., Gürtler M., Segel M., Van Dervort A., Ryu J.H., Peterson Q.P., Greiner D., Melton D.A. (2014). Generation of functional human pancreatic β cells in vitro. Cell.

[43] Nair G.G., Liu J.S., Russ H.A., Tran S., Saxton M.S., Chen R., Juang C., Li M.L., Nguyen V.Q., Giacometti S. (2019). Recapitulating endocrine cell clustering in culture promotes maturation of human stem-cell-derived β cells. Nat. Cell Biol..

[44] Bruin J.E., Erener S., Vela J., Hu X., Johnson J.D., Kurata H.T., Lynn F.C., Piret J.M., Asadi A., Rezania A. (2014). Characterization of polyhormonal insulin-producing cells derived in vitro from human embryonic stem cells. Stem Cell Res..

[45] Hrvatin S., O’Donnell C.W., Deng F., Millman J.R., Pagliuca F.W., DiIorio P., Rezania A., Gifford D.K., Melton D.A. (2014). Differentiated human stem cells resemble fetal, not adult, β cells. Proc. Natl. Acad. Sci. USA.

[46] Veres A., Faust A.L., Bushnell H.L., Engquist E.N., Kenty J.H.-R., Harb G., Poh Y.-C., Sintov E., Gürtler M., Pagliuca F.W. (2019). Charting cellular identity during human in vitro β-cell differentiation. Nature.

[47] Youngblood R.L., Sampson J.P., Lebioda K.R., Shea L.D. (2019). Microporous scaffolds support assembly and differentiation of pancreatic progenitors into β-cell clusters. Acta Biomater..

[48] Yoshihara E., O’Connor C., Gasser E., Wei Z., Oh T.G., Tseng T.W., Wang D., Cayabyab F., Dai Y., Yu R.T. (2020). Immune-evasive human islet-like organoids ameliorate diabetes. Nature.

[49] Gan W.J., Zavortink M., Ludick C., Templin R., Webb R., Webb R., Ma W., Poronnik P., Parton R.G., Gaisano H.Y. (2017). Cell polarity defines three distinct domains in pancreatic β-cells. J. Cell Sci..

[50] Almaça J., Weitz J., Rodriguez-Diaz R., Pereira E., Caicedo A. (2018). The Pericyte of the Pancreatic Islet Regulates Capillary Diameter and Local Blood Flow. Cell Metab..

[51] Takahashi Y., Sekine K., Kin T., Takebe T., Taniguchi H. (2018). Self-Condensation Culture Enables Vascularization of Tissue Fragments for Efficient Therapeutic Transplantation. Cell Rep..

[52] Takahashi Y., Takebe T., Taniguchi H. (2018). Methods for Generating Vascularized Islet-Like Organoids Via Self-Condensation. Curr. Protoc. Stem Cell Biol..

[53] Palikuqi B., Nguyen D.-H.T., Li G., Schreiner R., Pellegata A.F., Liu Y., Redmond D., Geng F., Lin Y., Gómez-Salinero J.M. (2020). Adaptable haemodynamic endothelial cells for organogenesis and tumorigenesis. Nature.

[54] Malin S.K., Finnegan S., Fealy C.E., Filion J., Rocco M.B., Kirwan J.P. (2014). β-Cell dysfunction is associated with metabolic syndrome severity in adults. Metab. Syndr. Relat. Disord..

[55] Talchai C., Xuan S., Lin H.V., Sussel L., Accili D. (2012). Pancreatic β cell dedifferentiation as a mechanism of diabetic β cell failure. Cell.

[56] Rui J., Deng S., Arazi A., Perdigoto A.L., Liu Z., Herold K.C. (2017). β Cells that Resist Immunological Attack Develop during Progression of Autoimmune Diabetes in NOD Mice. Cell Metab..

[57] Kulkarni R.N., Mizrachi E.-B., Ocana A.G., Stewart A.F. (2012). Human β-cell proliferation and intracellular signaling: Driving in the dark without a road map. Diabetes.

[58] Bernal-Mizrachi E., Kulkarni R.N., Scott D.K., Mauvais-Jarvis F., Stewart A.F., Garcia-Ocaña A. (2014). Human β-cell proliferation and intracellular signaling part 2: Still driving in the dark without a road map. Diabetes.

[59] Stewart A.F., Hussain M.A., García-Ocaña A., Vasavada R.C., Bhushan A., Bernal-Mizrachi E., Kulkarni R.N. (2015). Human β-cell proliferation and intracellular signaling: Part 3. Diabetes.

[60] Dor Y., Brown J., Martinez O.I., Melton D.A. (2004). Adult pancreatic beta-cells are formed by self-duplication rather than stem-cell differentiation. Nature.

[61] Wei P., Li L., Qi H., Zhou H.-X., Deng C.-Y., Li F.-R. (2012). Reversible immortalization of Nestin-positive precursor cells from pancreas and differentiation into insulin-secreting cells. Biochem. Biophys. Res. Commun..

[62] Van der Meulen T., Mawla A.M., DiGruccio M.R., Adams M.W., Nies V., Dólleman S., Liu S., Ackermann A.M., Cáceres E., Hunter A.E. (2017). Virgin Beta Cells Persist throughout Life at a Neogenic Niche within Pancreatic Islets. Cell Metab..

[63] Qadir M.M.F., Álvarez-Cubela S., Klein D., Lanzoni G., García-Santana C., Montalvo A., Pláceres-Uray F., Mazza E.M.C., Ricordi C., Inverardi L.A. (2018). P2RY1/ALK3-Expressing Cells within the Adult Human Exocrine Pancreas Are BMP-7 Expandable and Exhibit Progenitor-like Characteristics. Cell Rep..

[64] Loomans C.J.M., Williams Giuliani N., Balak J., Ringnalda F., van Gurp L., Huch M., Boj S.F., Sato T., Kester L., de Sousa Lopes S.M.C. (2018). Expansion of Adult Human Pancreatic Tissue Yields Organoids Harboring Progenitor Cells with Endocrine Differentiation Potential. Stem Cell Rep..

[65] Wang D., Wang J., Bai L., Pan H., Feng H., Clevers H., Zeng Y.A. (2020). Long-Term Expansion of Pancreatic Islet Organoids from Resident Procr+ Progenitors. Cell.

[66] Murphy M.P. (2009). How mitochondria produce reactive oxygen species. Biochem. J..

[67] Rani V., Deep G., Singh R.K., Palle K., Yadav U.C.S. (2016). Oxidative stress and metabolic disorders: Pathogenesis and therapeutic strategies. Life Sci..

[68] Vernier M., Dufour C.R., McGuirk S., Scholtes C., Li X., Bourmeau G., Kuasne H., Park M., St-Pierre J., Audet-Walsh E. (2020). Estrogen-related receptors are targetable ROS sensors. Genes Dev..

[69] Yoshihara E., Wei Z., Lin C.S., Fang S., Ahmadian M., Kida Y., Tseng T., Dai Y., Yu R.T., Liddle C. (2016). ERRγ Is Required for the Metabolic Maturation of Therapeutically Functional Glucose-Responsive β Cells. Cell Metab..

[70] Choi J., Oh T.G., Jung H.-W., Park K.-Y., Shin H., Jo T., Kang D.-S., Chanda D., Hong S., Kim J. (2022). Estrogen-Related Receptor γ Maintains Pancreatic Acinar Cell Function and Identity by Regulating Cellular Metabolism. Gastroenterology.

[71] Perelis M., Marcheva B., Ramsey K.M., Schipma M.J., Hutchison A.L., Taguchi A., Peek C.B., Hong H., Huang W., Omura C. (2015). Pancreatic β cell enhancers regulate rhythmic transcription of genes controlling insulin secretion. Science.

[72] Alvarez-Dominguez J.R., Donaghey J., Rasouli N., Kenty J.H.R., Helman A., Charlton J., Straubhaar J.R., Meissner A., Melton D.A. (2020). Circadian Entrainment Triggers Maturation of Human In Vitro Islets. Cell Stem Cell.

[73] Asrih M., Jornayvaz F.R. (2015). Metabolic syndrome and nonalcoholic fatty liver disease: Is insulin resistance the link?. Mol. Cell. Endocrinol..

[74] Loomba R., Friedman S.L., Shulman G.I. (2021). Mechanisms and disease consequences of nonalcoholic fatty liver disease. Cell.

[75] Angulo P., Kleiner D.E., Dam-Larsen S., Adams L.A., Bjornsson E.S., Charatcharoenwitthaya P., Mills P.R., Keach J.C., Lafferty H.D., Stahler A. (2015). Liver Fibrosis, but No Other Histologic Features, Is Associated with Long-term Outcomes of Patients with Nonalcoholic Fatty Liver Disease. Gastroenterology.

[76] Hagström H., Nasr P., Ekstedt M., Hammar U., Stål P., Hultcrantz R., Kechagias S. (2017). Fibrosis stage but not NASH predicts mortality and time to development of severe liver disease in biopsy-proven NAFLD. J. Hepatol..

[77] Bedossa P. (2014). Utility and appropriateness of the fatty liver inhibition of progression (FLIP) algorithm and steatosis, activity, and fibrosis (SAF) score in the evaluation of biopsies of nonalcoholic fatty liver disease. Hepatology.

[78] Singh S., Allen A.M., Wang Z., Prokop L.J., Murad M.H., Loomba R. (2015). Fibrosis progression in nonalcoholic fatty liver vs nonalcoholic steatohepatitis: A systematic review and meta-analysis of paired-biopsy studies. Clin. Gastroenterol. Hepatol..

[79] Friedman S.L. (2003). Liver fibrosis -- From bench to bedside. J. Hepatol..

[80] Bataller R., Brenner D.A. (2005). Liver fibrosis. J. Clin. Investig..

[81] Tsuchida T., Friedman S.L. (2017). Mechanisms of hepatic stellate cell activation. Nat. Rev. Gastroenterol. Hepatol..

[82] Cheng D., Chai J., Wang H., Fu L., Peng S., Ni X. (2021). Hepatic macrophages: Key players in the development and progression of liver fibrosis. Liver Int..

[83] Wells R.G. (2014). The portal fibroblast: Not just a poor man’s stellate cell. Gastroenterology.

[84] Sato K., Marzioni M., Meng F., Francis H., Glaser S., Alpini G. (2019). Ductular Reaction in Liver Diseases: Pathological Mechanisms and Translational Significances. Hepatology.

[85] Huch M., Dorrell C., Boj S.F., van Es J.H., Li V.S.W., van de Wetering M., Sato T., Hamer K., Sasaki N., Finegold M.J. (2013). In vitro expansion of single Lgr5+ liver stem cells induced by Wnt-driven regeneration. Nature.

[86] Huch M., Gehart H., van Boxtel R., Hamer K., Blokzijl F., Verstegen M.M.A., Ellis E., van Wenum M., Fuchs S.A., de Ligt J. (2015). Long-term culture of genome-stable bipotent stem cells from adult human liver. Cell.

[87] Takebe T., Sekine K., Enomura M., Koike H., Kimura M., Ogaeri T., Zhang R.-R., Ueno Y., Zheng Y.-W., Koike N. (2013). Vascularized and functional human liver from an iPSC-derived organ bud transplant. Nature.

[88] Takebe T., Sekine K., Kimura M., Yoshizawa E., Ayano S., Koido M., Funayama S., Nakanishi N., Hisai T., Kobayashi T. (2017). Massive and Reproducible Production of Liver Buds Entirely from Human Pluripotent Stem Cells. Cell Rep..

[89] Wu F., Wu D., Ren Y., Huang Y., Feng B., Zhao N., Zhang T., Chen X., Chen S., Xu A. (2019). Generation of hepatobiliary organoids from human induced pluripotent stem cells. J. Hepatol..

[90] Ouchi R., Togo S., Kimura M., Shinozawa T., Koido M., Koike H., Thompson W., Karns R.A., Mayhew C.N., McGrath P.S. (2019). Modeling Steatohepatitis in Humans with Pluripotent Stem Cell-Derived Organoids. Cell Metab..

[91] Ramli M.N.B., Lim Y.S., Koe C.T., Demircioglu D., Tng W., Gonzales K.A.U., Tan C.P., Szczerbinska I., Liang H., Soe E.L. (2020). Human Pluripotent Stem Cell-Derived Organoids as Models of Liver Disease. Gastroenterology.

[92] Shinozawa T., Kimura M., Cai Y., Saiki N., Yoneyama Y., Ouchi R., Koike H., Maezawa M., Zhang R.-R., Dunn A. (2021). High-Fidelity Drug-Induced Liver Injury Screen Using Human Pluripotent Stem Cell-Derived Organoids. Gastroenterology.

[93] Teufel A., Itzel T., Erhart W., Brosch M., Wang X.Y., Kim Y.O., von Schönfels W., Herrmann A., Brückner S., Stickel F. (2016). Comparison of Gene Expression Patterns between Mouse Models of Nonalcoholic Fatty Liver Disease and Liver Tissues from Patients. Gastroenterology.

[94] Lyall M.J., Cartier J., Thomson J.P., Cameron K., Meseguer-Ripolles J., O’Duibhir E., Szkolnicka D., Villarin B.L., Wang Y., Blanco G.R. (2018). Modelling non-alcoholic fatty liver disease in human hepatocyte-like cells. Philos. Trans. R. Soc. Lond. B Biol. Sci..

[95] Sinton M.C., Meseguer-Ripolles J., Lucendo-Villarin B., Wernig-Zorc S., Thomson J.P., Carter R.N., Lyall M.J., Walker P.D., Thakker A., Meehan R.R. (2021). A human pluripotent stem cell model for the analysis of metabolic dysfunction in hepatic steatosis. iScience.

[96] McCarron S., Bathon B., Conlon D.M., Abbey D., Rader D.J., Gawronski K., Brown C.D., Olthoff K.M., Shaked A., Raabe T.D. (2021). Functional Characterization of Organoids Derived from Irreversibly Damaged Liver of Patients with NASH. Hepatology.

[97] Kisseleva T., Brenner D. (2021). Molecular and cellular mechanisms of liver fibrosis and its regression. Nat. Rev. Gastroenterol. Hepatol..

[98] Sanyal A.J., Mofrad P.S., Contos M.J., Sargeant C., Luketic V.A., Sterling R.K., Stravitz R.T., Shiffman M.L., Clore J., Mills A.S. (2004). A pilot study of vitamin E versus vitamin E and pioglitazone for the treatment of nonalcoholic steatohepatitis. Clin. Gastroenterol. Hepatol..

[99] Wei J., Qiu D.K., Ma X. (2009). Bile acids and insulin resistance: Implications for treating nonalcoholic fatty liver disease. J. Dig. Dis..

[100] Harrison S.A., Abdelmalek M.F., Caldwell S., Shiffman M.L., Diehl A.M., Ghalib R., Lawitz E.J., Rockey D.C., Schall R.A., Jia C. (2018). Simtuzumab Is Ineffective for Patients with Bridging Fibrosis or Compensated Cirrhosis Caused by Nonalcoholic Steatohepatitis. Gastroenterology.

[101] Meirelles Júnior R.F., Salvalaggio P., Rezende M.B.d., Evangelista A.S., Guardia B.D., Matielo C.E.L., Neves D.B., Pandullo F.L., Felga G.E.G., Alves J.A.d.S. (2015). Liver transplantation: History, outcomes and perspectives. Einstein.

[102] Fausto N., Campbell J.S., Riehle K.J. (2006). Liver regeneration. Hepatology.

[103] Michalopoulos G.K., DeFrances M.C. (1997). Liver regeneration. Science.

[104] Michalopoulos G.K., Khan Z. (2015). Liver Stem Cells: Experimental Findings and Implications for Human Liver Disease. Gastroenterology.

[105] Tarlow B.D., Pelz C., Naugler W.E., Wakefield L., Wilson E.M., Finegold M.J., Grompe M. (2014). Bipotential adult liver progenitors are derived from chronically injured mature hepatocytes. Cell Stem Cell.

[106] Hattoum A., Rubin E., Orr A., Michalopoulos G.K. (2013). Expression of hepatocyte epidermal growth factor receptor, FAS and glypican 3 in EpCAM-positive regenerative clusters of hepatocytes, cholangiocytes, and progenitor cells in human liver failure. Hum. Pathol..

[107] Peng J., Li F., Wang J., Wang C., Jiang Y., Liu B., He J., Yuan K., Pan C., Lin M. (2022). Identification of a rare Gli1+ progenitor cell population contributing to liver regeneration during chronic injury. Cell Discov..

[108] Terai S., Tsuchiya A., Watanabe Y., Takeuchi S. (2021). Transition of clinical and basic studies on liver cirrhosis treatment using cells to seek the best treatment. Inflamm. Regen..

[109] Uderhardt S., Martins A.J., Tsang J.S., Lämmermann T., Germain R.N. (2019). Resident Macrophages Cloak Tissue Microlesions to Prevent Neutrophil-Driven Inflammatory Damage. Cell.

[110] Tacke F., Zimmermann H.W. (2014). Macrophage heterogeneity in liver injury and fibrosis. J. Hepatol..

[111] Takeuchi S., Tsuchiya A., Iwasawa T., Nojiri S., Watanabe T., Ogawa M., Yoshida T., Fujiki K., Koui Y., Kido T. (2021). Small extracellular vesicles derived from interferon-γ pre-conditioned mesenchymal stromal cells effectively treat liver fibrosis. NPJ Regen. Med..

[112] Kimura M., Iguchi T., Iwasawa K., Dunn A., Thompson W.L., Yoneyama Y., Chaturvedi P., Zorn A.M., Wintzinger M., Quattrocelli M. (2022). En masse organoid phenotyping informs metabolic-associated genetic susceptibility to NASH. Cell.

[113] Collin de l’Hortet A., Takeishi K., Guzman-Lepe J., Morita K., Achreja A., Popovic B., Wang Y., Handa K., Mittal A., Meurs N. (2019). Generation of Human Fatty Livers Using Custom-Engineered Induced Pluripotent Stem Cells with Modifiable SIRT1 Metabolism. Cell Metab..

[114] Hendriks D., Brouwers J.F., Hamer K., Geurts M.H., Luciana L., Massalini S., López-Iglesias C., Peters P.J., Rodríguez-Colman M.J., Chuva de Sousa Lopes S. (2023). Engineered human hepatocyte organoids enable CRISPR-based target discovery and drug screening for steatosis. Nat. Biotechnol..

[115] Aisenbrey E.A., Murphy W.L. (2020). Synthetic alternatives to Matrigel. Nat. Rev. Mater..

[116] Gjorevski N., Sachs N., Manfrin A., Giger S., Bragina M.E., Ordóñez-Morán P., Clevers H., Lutolf M.P. (2016). Designer matrices for intestinal stem cell and organoid culture. Nature.

[117] Ranga A., Gobaa S., Okawa Y., Mosiewicz K., Negro A., Lutolf M.P. (2014). 3D niche microarrays for systems-level analyses of cell fate. Nat. Commun..

[118] Raza A., Ki C.S., Lin C.-C. (2013). The influence of matrix properties on growth and morphogenesis of human pancreatic ductal epithelial cells in 3D. Biomaterials.

[119] Hogrebe N.J., Augsornworawat P., Maxwell K.G., Velazco-Cruz L., Millman J.R. (2020). Targeting the cytoskeleton to direct pancreatic differentiation of human pluripotent stem cells. Nat. Biotechnol..

[120] Correia Carreira S., Begum R., Perriman A.W. (2020). 3D Bioprinting: The Emergence of Programmable Biodesign. Adv. Healthc. Mater..

[121] Chakraborty J., Chawla S., Ghosh S. (2022). Developmental biology-inspired tissue engineering by combining organoids and 3D bioprinting. Curr. Opin. Biotechnol..

[122] Beattie G.M., Rubin J.S., Mally M.I., Otonkoski T., Hayek A. (1996). Regulation of proliferation and differentiation of human fetal pancreatic islet cells by extracellular matrix, hepatocyte growth factor, and cell-cell contact. Diabetes.

[123] Gage B.K., Webber T.D., Kieffer T.J. (2013). Initial cell seeding density influences pancreatic endocrine development during in vitro differentiation of human embryonic stem cells. PLoS ONE.

[124] Memon B., Karam M., Al-Khawaga S., Abdelalim E.M. (2018). Enhanced differentiation of human pluripotent stem cells into pancreatic progenitors co-expressing PDX1 and NKX6.1. Stem Cell Res. Ther..

[125] Bernal P.N., Delrot P., Loterie D., Li Y., Malda J., Moser C., Levato R. (2019). Volumetric Bioprinting of Complex Living-Tissue Constructs within Seconds. Adv. Mater..

[126] Daly A.C., Davidson M.D., Burdick J.A. (2021). 3D bioprinting of high cell-density heterogeneous tissue models through spheroid fusion within self-healing hydrogels. Nat. Commun..

[127] Liu H., Wang Y., Wang H., Zhao M., Tao T., Zhang X., Qin J. (2020). A Droplet Microfluidic System to Fabricate Hybrid Capsules Enabling Stem Cell Organoid Engineering. Adv. Sci..

[128] Koike H., Iwasawa K., Ouchi R., Maezawa M., Giesbrecht K., Saiki N., Ferguson A., Kimura M., Thompson W.L., Wells J.M. (2019). Modelling human hepato-biliary-pancreatic organogenesis from the foregut-midgut boundary. Nature.

[129] Vunjak-Novakovic G., Ronaldson-Bouchard K., Radisic M. (2021). Organs-on-a-chip models for biological research. Cell.

[130] Tao T., Deng P., Wang Y., Zhang X., Guo Y., Chen W., Qin J. (2022). Microengineered Multi-Organoid System from hiPSCs to Recapitulate Human Liver-Islet Axis in Normal and Type 2 Diabetes. Adv. Sci..

[131] Abou Ziki M.D., Mani A. (2016). Metabolic syndrome: Genetic insights into disease pathogenesis. Curr. Opin. Lipidol..

[132] Hoffmann K., Mattheisen M., Dahm S., Nürnberg P., Roe C., Johnson J., Cox N.J., Wichmann H.E., Wienker T.F., Schulze J. (2007). A German genome-wide linkage scan for type 2 diabetes supports the existence of a metabolic syndrome locus on chromosome 1p36.13 and a type 2 diabetes locus on chromosome 16p12.2. Diabetologia.

[133] Romeo S., Kozlitina J., Xing C., Pertsemlidis A., Cox D., Pennacchio L.A., Boerwinkle E., Cohen J.C., Hobbs H.H. (2008). Genetic variation in PNPLA3 confers susceptibility to nonalcoholic fatty liver disease. Nat. Genet..

[134] Liang G., Zhang Y. (2013). Genetic and epigenetic variations in iPSCs: Potential causes and implications for application. Cell Stem Cell.

